# Development of anti-membrane type 1-matrix metalloproteinase nanobodies as immunoPET probes for triple negative breast cancer imaging

**DOI:** 10.3389/fmed.2022.1058455

**Published:** 2022-11-24

**Authors:** Francisca Mulero, Marta Oteo, Guillermo Garaulet, Natalia Magro, Lluvia Rebollo, Guillermo Medrano, Clara Santiveri, Eduardo Romero, Ricela E. Sellek, Yago Margolles, Ramón Campos-Olivas, Alicia G. Arroyo, Luis Angel Fernández, Miguel Angel Morcillo, Jorge L. Martínez-Torrecuadrada

**Affiliations:** ^1^Molecular Imaging Unit, Spanish National Cancer Research Centre (CNIO), Madrid, Spain; ^2^Medical Applications of Ionizing Radiations Unit, Centro de Investigaciones Energéticas, Medioambientales y Tecnológicas (CIEMAT), Madrid, Spain; ^3^Protein Production Unit, Spanish National Cancer Research Centre (CNIO), Madrid, Spain; ^4^Spectroscopy and Nuclear Magnetic Resonance Unit, Spanish National Cancer Research Centre (CNIO), Madrid, Spain; ^5^Department of Microbial Biotechnology, Centro Nacional de Biotecnología, (CNB-CSIC), Madrid, Spain; ^6^Department of Molecular Biomedicine, Centro de Investigaciones Biológicas Margarita Salas (CIB-CSIC), Madrid, Spain

**Keywords:** immunoPET, MT1-MMP, nanobodies, gallium-68, TNBC (triple negative breast cancer)

## Abstract

Triple-negative breast cancer (TNBC) is characterized by aggressiveness and high rates of metastasis. The identification of relevant biomarkers is crucial to improve outcomes for TNBC patients. Membrane type 1-matrix metalloproteinase (MT1-MMP) could be a good candidate because its expression has been reported to correlate with tumor malignancy, progression and metastasis. Moreover, single-domain variable regions (VHHs or Nanobodies) derived from camelid heavy-chain-only antibodies have demonstrated improvements in tissue penetration and blood clearance, important characteristics for cancer imaging. Here, we have developed a nanobody-based PET imaging strategy for TNBC detection that targets MT1-MMP. A llama-derived library was screened against the catalytic domain of MT1-MMP and a panel of specific nanobodies were identified. After a deep characterization, two nanobodies were selected to be labeled with gallium-68 (^68^Ga). ImmunoPET imaging with both ([^68^Ga]Ga-NOTA-3TPA14 and [^68^Ga]Ga-NOTA-3CMP75) in a TNBC mouse model showed precise tumor-targeting capacity *in vivo* with high signal-to-background ratios. (^68^Ga)Ga-NOTA-3CMP75 exhibited higher tumor uptake compared to (^68^Ga)Ga-NOTA-3TPA14. Furthermore, imaging data correlated perfectly with the immunohistochemistry staining results. In conclusion, we found a promising candidate for nanobody-based PET imaging to be further investigated as a diagnostic tool in TNBC.

## Introduction

Breast cancer is the most common malignancy diagnosed in women worldwide, contributing 24.5% of the total number of new cases diagnosed in 2020 and a global cancer incidence of 11.7% for both sexes combined (1). Currently, the treatment of patients with triple-negative breast cancer (TNBC) is the biggest challenge. TNBC represents 15–25% of all breast cancers (2) and is characterized by the lack of estrogen receptor (ER), progesterone receptor (PR) and human epidermal growth factor receptor (HER2). TNBCs tend to behave more aggressively, have high rates of metastasis in secondary organs like brain, bone and lungs and frequently are histologic high-grade tumors associated with higher rates of relapse and mortality (3). Metastasis is the leading cause of death for the vast majority of TNBC patients and non-effective targeted therapies has been developed yet such as those for luminal and HER2 breast cancer subtypes. Metastatic spread of cancer is a complex, and poorly understood process that involves cancer cells acquiring invasive properties to degrade basement membranes and spread to distal tissues *via* blood or lymphatic vessels (4). In this regard, membrane type 1-matrix metalloproteinase (MT1-MMP), a zinc-dependent membrane-anchored matrix metalloproteinase, has been described playing an important role involved in invasive tumor growth, progression and metastasis in triple negative breast cancer (5) and in other metastatic cancer models (6–8). In normal tissues, it is expressed at low levels in only certain types of cells such as fibroblasts or endothelial cells, among others (9) although its physiological functions are not yet fully understood; whereas MT1-MMP is widely expressed during embryonic development and pathological conditions (10). This protein has been also found to be associated with high cancer aggressiveness and poor prognosis (11) and is generally overexpressed in high grade TNBC (7, 12, 13). Besides, MT1-MMP expression is also related with lung and brain metastases (5, 14). These features encourage exploring MT1-MMP protein as a candidate biomarker for diagnostic purposes of TNBC with the aim of improving the clinical management of these patients.

Among several non-invasive diagnostic methods, conventional ^18^F-FDG PET imaging has been proven to be useful for the detection of TNBC (15), however, a poor specificity is a major concern that limits the application of ^18^F-FDG in clinical setting, since can only illustrate the metabolic status and fail to provide tumor information at cellular/molecular level. Therefore, the development of novel targeting agents for TNBC is highly needed to guide tumor diagnosis, prognosis prediction and treatment monitoring. A promising option to improve diagnostic imaging would be the immunoPET, which combines the high sensitivity and quantitative capabilities of PET with the specificity and selectivity of antibodies to bind a specific biomarker (16). In previous works, we have developed antibody-based approaches for PET imaging of MT1-MMP in glioma and pancreatic cancer based on the murine anti-MT1-MMP monoclonal antibody LEM2/15 (17, 18) illustrating the high potential of MT1-MMP as imaging-based biomarker for detection of several cancer types. However, the major drawbacks in immunoPET imaging with full-length antibodies are the relative slow pharmacokinetics and their prolonged circulation half-life due to their high molecular weight; therefore, PET imaging used to be performed several days after injection leading to a prolonged circulating radiation increasing the background and worsen the image quality. As alternative, variable heavy regions derived from camelid heavy-chain-only antibodies (VHHs or Nanobodies) (19) can overcome the limitations of intact antibodies for PET imaging (20). Their small size (about 15 KDa) allows much deeper tissue penetration and faster blood clearance by renal excretion route increasing tumor to background ratio and image quality. Moreover, this shorter half-life can match with rapidly decaying radionuclides such as ^68^Ga (T_1/2_, 67.7 min) permitting fast imaging protocols. In addition, ^68^Ga has the advantage of being synthesized by a cyclotron-independent ^68^Ge/^68^Ga generator system that can be accessible on site, immediately in any PET center (21).

Owing to their unique and well-characterized properties, nanobodies have become extremely useful tools in diagnostics, therapeutics and research; especially in TNBC (see (22) for review). Here, we report the generation and characterization of MT1-MMP-targeting nanobodies from a llama immunized with the catalytic domain of MT1-MMP at the VIB Nanobody Core (Vrije Universiteit Brussel), and the development of ^68^Ga-labeled nanobody tracers to specifically detect MT1-MMP expression in a TNBC xenograft model for imaging diagnostic purposes by ImmunoPET.

## Materials and methods

### Mice and cell lines

Human breast cell line MDA-MB-231 was obtained from the European Collection of Authenticated Cell Cultures (ECACC) and cultured at 37°C in 5% CO_2_ humidity according to standard mammalian tissue culture protocols in Dulbecco’s Modified Eagle’s Medium + GlutaMax (Gibco, Waltham, MA) containing 10% fetal bovine serum (FBS) (Gibco, Waltham, MA) and 100 units/ml penicillin/100 μg/ml streptomycin (Gibco, Waltham, MA).

Athymic nude female mice (AthymicNude-Foxn1nu, ENVIGO, Indianapolis, IN) of 5 weeks of age were used to develop the breast cancer xenograft model.

### Purification of catalytic domain of membrane type 1-matrix metalloproteinase

The catalytic domain of human MT1-MMP (residues 119-290) (CAT-MT1-MMP) was expressed from pET3a (Novagen, Darmstadt) as an N-terminal 6XHis tagged protein. Briefly, CAT-MT1-MMP was expressed in BL21 (DE3) (Invitrogen, Waltham, MA) *E. coli* cells grown in lysogeny broth (LB) media plus ampicillin by induction with 0.5 mM isopropyl thio-ß-D-galactopyranoside (IPTG), 4 h at 37°C. Cells were resuspended in lysis buffer (50 mM Tris-HCl, 100 mM NaCl, 2 mM EDTA, 5 mM β-mercaptoethanol, 0.1% Brij-35, pH 7.5) followed by sonication and centrifugation. The pellet containing insoluble CAT-MT1-MMP inclusion bodies (IBs) was washed three times with washing buffer (50 mM Tris-HCl, 2 M NaCl, 2 mM EDTA, 5 mM β-mercaptoethanol, 0.1% Brij-35, pH 8.0). Isolated CAT-MT1-MMP IBs were solubilized in denaturing buffer (6 M Guanidinine hydrochoride, 50 mM Tris-HCl, 300 mM NaCl, 10 mM Imidazole, 5 mM 2-mercaptoethanol, pH 8.0) at 4°C overnight. Solubilized CAT-MT1-MMP was subjected to an immobilized metal ion affinity chromatography (IMAC) on a HisTrap column (Cytiva, Uppsala) using an ÄKTAgo (Cytiva, Uppsala). A gradual refolding of the bound protein was performed on-column using a linear urea gradient from 8.0 to 0 M in 50 mM Tris-HCl, 300 mM NaCl, 10 mM Imidazole, 5 mM 2-mercaptoethanol, pH 8.0, and then the recombinant CAT-MT1-MMP was eluted with an imidazole gradient up to 0.5 M. The eluted proteins were further purified by gel-filtration chromatography using a HiLoad 16/60 Superdex 75 column (Cytiva, Uppsala) attached with ÄKTAFPLC system (Cytiva, Uppsala). The fractions were concentrated in a Vivaspin 15R, 5 KDa molecular weight cut-off (MWCO) device (Sartorius, Stonehouse) and flash-frozen in liquid nitrogen.

### Membrane type 1-matrix metalloproteinase-specific nanobody generation

Immunization, construction of the VHH libraries and panning were performed by the Vlaams Instituut voor Biotechnologie (VIB) Nanobody Core (Vrije Universiteit Brussel, Belguim) as previously described (23). A llama was subcutaneously injected on days 0, 7, 14, 21, 28, and 35, each time with about 100 μg of recombinant catalytic domain of MT1-MMP using Gerbu P adjuvant (Gerbu Biotechnik GmbH, Heidelberg). On day 40 (5 days after last immunization), about 100 ml anticoagulated blood was collected from the llama for peripheral blood lymphocytes (PBLs) preparation. VHH phage-display library was generated by RT-PCR on mRNA of PBLs isolated from the immunized llama and cloned in the vector pMECS. The repertoire of the VHH library was displayed on phages and panned for four rounds against CAT-MT1-MMP directly adsorbed in wells of microtiter plates. Next, colonies were randomly selected and their periplasmic proteins were tested for CAT-MT1-MMP specificity by ELISA. Positive clones were grown to prepare phagemide DNA from which the corresponding nanobody inserts were then sequenced.

Recombinant pMECS plasmids encoding for selected nanobodies were transformed into non-suppressor *E. coli* WK6 strain. The WK6 cells were cultured in TB medium and induced with 1 mM IPTG overnight at 28°C. The periplasmic proteins were released by osmotic shock from the cells and 6xHis-tagged nanobodies were purified by IMAC using HisTrap columns (Cytiva, Uppsala) in a ÄKTAgo system (Cytiva, Uppsala) followed by a size-exclusion chromatography on a Hiload 16/60 Superdex 75 Column (Cytiva, Uppsala) equilibrated in PBS buffer and coupled to an ÄKTA FPLC system (Cytiva, Uppsala). Peak fractions were tested by SDS-PAGE followed by Coomassie blue staining, pooled and concentrated using a Vivaspin 15R device (Sartorius, Stonehouse). Finally, purified nanobodies were flash-frozen in liquid nitrogen and stored at −80°C. Also, a non-targeting Nb was purified in the same manner to be used as negative control for *in vivo* experiments, cAb-Lys3 which is a specific nanobody against lysozyme (24).

### Specificity by monoclonal phage ELISA

Individual phages were subjected to phage ELISA to investigate cross reactivity with other closely related members of the membrane-type matrix metalloproteinase subgroup, MT2-MMP and MT3-MMP. For this purpose, selected individual colonies of *E. coli* TG1 harboring phagemids were grown in 2 × YT medium overnight at 30°C in a 96-well plate (Nunclon, Roskilde). Then, the overnight culture for each clone was diluted 100-fold into fresh 2xYT medium and incubated at 37°C for 2 h. The cultures were infected with ∼10^9^ PFU of VCS-M13 (KmR; Stratagene, San Diego, CA) helper phages and grown overnight at 30°C. Finally, the culture supernatant of each well was used for ELISA. MaxiSorp 96-well plates (Nunc, Roskilde) were coated with CAT-MT1-MMP, MT2-MMP [NS0-derived fragment from Glu47 to Pro565 (Arg128Pro; Arg129Gly), R&D Systems, Minneapolis, MN] or MT3-MMP [*E. coli*-derived fragment from Ala32 to Gly291 (Ile152Asn); R&D Systems, Minneapolis, MN] at 4°C overnight. Bovine serum albumin (BSA, Roche, Basilea) was used as a negative control for detection. The supernatants containing the corresponding phages were applied to the plates and bound phages were detected with an anti-M13-HRP mAb (GE Healthcare, Uppsala) and o-phenylenediamine (OPD, Sigma, Saint Louis, MO) as substrate. The OD490 was determined using a microplate reader (iMark ELISA plate reader, Bio-Rad, Hercules, CA).

### Size-exclusion chromatography combined with multiangle light scattering

3TPA14 and 2CMP75 samples, 200 μl at 2.8 mg/ml and 100 μl at 2.6 mg/ml, respectively, were injected in a Superdex 200 Increase 10/300 column (Cytiva, Uppsala) equilibrated in 0.1 μm-filtered HBS-EP + buffer (10 mM HEPES pH 7.3, 150 mM NaCl, 3 mM EDTA and 0.05% surfactant P20) and connected to an AKTA Purifier equipment (GE Healthcare, Uppsala). The chromatographic eluent was monitored by three consecutive detectors in series: (1) a multi-wavelength UV-Vis absorbance Monitor UV-900 (GE Healthcare, Uppsala) detector of the AKTA system, (2) a light scattering DAWN Heleos 8 + (Wyatt Technology, Santa Barbara, CA) detector and (3) an Optilab T-rEX (Wyatt Technology, Santa Barbara, CA) differential refractive index detector. The column was equilibrated overnight in running buffer at 0.1 ml/min flow to obtain stable base lines before data collection. After that, all the experiments were performed at 0.5 ml/min flow and room temperature. Before running test samples, a control run with BSA, a well-characterized monodisperse sample, was carried out to set the alignment and band broadening parameters and the normalization coefficients of the MALS detectors necessary for data analysis. Data collection and analysis were performed using UNICORN 5.10 (GE Healthcare, Uppsala) and ASTRA 6.0.3 (Wyatt Technology, Santa Barbara, CA) software packages.

### Nano differential scanning fluorimetry

CAT-MT1-MMP, 3TPA14, and 2CMP75 thermal shift assays were performed with a Tycho NT.6 instrument (NanoTemper Technologies, Munich). 10 μl protein samples, corresponding to the eluted fractions after size-exclusion chromatography combined with multiangle light scattering (SEC-MALS), were heated in a glass capillary from 35–95°C at a rate of 30°C/min. The intrinsic fluorescence (mostly arising from tryptophan and tyrosine amino acids) was recorded at 330 and 350 nm and the inflection temperature (Ti) was determined from the maximum of the first derivative of the fluorescence intensity ratio (F350/F330) with the Tycho NT.6 software.

### Surface plasmon resonance

All the SPR experiments were performed on a Biacore X100 instrument (GE Healthcare, Uppsala) at 25°C. Commercial NS0-derived extracellular domain (ed), from Ala21-Ser538, with an Arg108Pro mutation, of MT1-MMP (R&D Systems, Minneapolis, MN), at 317 μg/ml in 25 mM sodium acetate pH 5.0, 1 M NaCl, 5 mM CaCl_2_ and 20% (v/v) glycerol buffer, was 5-fold diluted in immobilization buffer (25 mM sodium acetate pH 5.0, 200 mM NaCl and 0.05% P20) and immobilized by standard amine coupling on a CM5 sensor chip (Cytiva, Uppsala). The active flow cell of the sensor chip was first treated with a fresh mixture of 0.4 M 1-(3-(dimethylamino) propyl)-ethylcarbodiimide (EDC)/0.1 M *N*-hydroxysuccimide (NHS) 1:1 in water (7 min injection at 10 μl/min flow rate). Then, edMT1-MMP (63 μg/ml) was injected for 16 min at 5 μl/min and, finally, the unreacted NHS-esters were deactivated by injecting 1 M ethanolamine-HCl pH 8.5 for 7 min at 10 μl/min, reaching a final immobilization level of edMT1-MMP of 104 RU. The reference flow cell was subjected to an equivalent treatment with EDC/NHS and ethanolamine but without flowing the protein.

Binding experiments were carried out using HBS-EP + buffer as running buffer. Each nanobody was tested in the concentration range of 16–250 nM (with 62 nM, 125 and 250 nM measurements performed in duplicate) and samples were injected for 3 min at a flow rate of 30 μl/min and dissociated for 30 min (no regeneration solution was needed). Solvent-correction and kinetic analysis of the sensorgrams were done with the BIAevaluation software (GE Healthcare, Uppsala) by applying a 1:1 binding model.

To test if the two antibodies compete for the same MT1-MMP epitope, we performed individual injections (3 min at 30 μl/min) of 3TPA14 (at 1.6 and 1 μM), 3CMP75 (at 3.6 and 1 μM) and mixtures at those same concentrations. The 1.6 and 3.6 μM concentrations of 3TPA14 and 3CMP75 were selected to match 100 × K_D_ values of both nanobodies, as determined by kinetic analysis of individual titrations.

### Radiolabeling of nanobodies

For conjugation, 2 mg of each nanobody in 1 ml of solution at pH 8.9–9.1, adjusted with 0.1 M Na_2_CO_3_, were mixed with p-SCN-Bn-NOTA (Macrocyclics, Plano, TX) and dissolved in DMSO at a final concentration 3.5 mM and at a molar ratio of 1:5. The reaction was incubated at 37°C for 90 min at 550 rpm. Non-conjugated chelator was removed using a PD-10 desalting column (Cytiva, Uppsala) equilibrated with 5 mg/ml gentisic acid in 0.25 M sodium acetate trihydrate (pH 5.4–5.6). Radiolabelling of NOTA-Nanobody with ^68^Ga was performed using fractionated elution from a ^68^Ge/^68^Ga-generator, based on nano-SnO_2_ and developed at CIEMAT (21), with 4 ml of 1 M HCl (VWR, Radnor, PA). Two vials containing 1 and 3 ml of eluate were collected and measured in a dose calibrator (IBC, Veenstra Instruments, Joure). The highest amount of activity, which contained approximately 90% of total elutable ^68^Ga (370–555 MBq, 1 ml), was placed in a 5 ml screw cap tube (Eppendorf, Hamburg). Next, 290 μl of 1 M HEPES sodium salt (Sigma-Aldrich, Saint Louis, MO) were added and finally 710 μl of NOTA-Nanobody (1 mg/ml) were added as well. The pH was checked to be at 3.5–5.0 using pH-indicator strips (Merck) and immediately the mixture was incubated at 30°C for 60 min at 550 rpm using a Thermomixer (Eppendorf, Hamburg). Finally, the reaction mixture was loaded onto a previously equilibrated PD-10 column and eluted with PBS into fractions of 500 μl. After purification, the collected fractions were measured in a dose calibrator. Radiochemical yield was determined as % of starting activity recovered after PD-10 purification. An aliquot was taken to analyse the radiochemical purity of the final product by radio-HPLC in a Jasco HPLC system equipped with a photodiode array UV-detector MD-4015 (Jasco, Easton, MD), a radioactivity detector LB 500 HERM (Berthold, Bad Wildbad), and a gel filtration comatography BioSep Sec-s2000 145 Å LC Column (5 μm, 300 × 7.8 mm).

### Reactivity of the anti-membrane type 1-matrix metalloproteinase nanobodies by ELISA

Enzyme-linked immunosorbent assay plates (MaxiSorp™, Sigma-Aldrich, Saint Louis, MO) were coated overnight at 4°C with CAT-MT1-MMP or an irrelevant recombinant protein as a negative control. Wells were blocked by 3% Bovine Serum Albumin in PBS at room temperature for 1 h. Next, serial dilutions of MT1-MMP nanobodies in blocking buffer were added and incubated for 90 min at 37°C. Bound nanobodies were detected by incubation with HRP-conjugated anti-HA (clone 6E2) mouse mAb (Cell Signaling, Danvers, MA) at room temperature for 1.15 h and TMB (Sigma-aldrich, Saint Louis, MO) as substrate. The reaction was stopped by adding 1 M sulfuric acid (Sigma-Aldrich, Saint Louis, MO) and the OD450 was measured (Fluostar Omega, BMG-Labtech, Ortenberg).

### Animal model

The high expression of MT1-MMP characterizes the triple negative MDA-MB-231 breast cancer cell line (12, 25–27). According to this, a triple negative breast cancer heterotopic xenograft model was generated by inoculating subcutaneously 3 × 10^6^ of MDA-MB-231 cells in 100 μl of a 1:1 mix of PBS (Gibco, Waltham, MA) with Matrigel (Corning, Corning, NY) into the right upper flank of 5 weeks athymic nude female mice. Tumors were allowed to develop until palpable prior immunoPET scan. Mice were sacrificed when the tumor mass reached a maximum size of 1,500 mm^3^ or tumor ulceration was observed or mice were symptomatic including signs of lethargy, poor grooming, weight loss and hunching.

All animal experimental procedures were performed following protocols approved by the Local Animal Ethical Committee; in strict adherence to the guidelines stated in the International Guiding Principles for Biomedical Research Involving Animals, established by the Council for International Organizations of Medical Sciences (CIOMS). All animal experimental procedures had also been approved by the Ethical Competent Authority (project PROEX 294.8/20).

### Biodistribution and pharmacokinetic studies using PET/CT imaging

For biodistribution, PET image acquisition was performed on a small-animal Super Argus 3r PET-CT scanner (SEDECAL, Madrid, Spain). Mice were anesthetized through inhalation of 2–2.5% isofluorane in 1 L of oxygen and were injected *via* the tail vein with the ^68^Ga radiolabeled-nanobodies. Dynamic PET imaging was performed over 60 min after the injection of [^68^Ga]Ga-NOTA-3TPA14 (2.2 ± 0.8 MBq; 19.7 ± 4.2 μg; *n* = 8), [^68^Ga]Ga-NOTA-3CMP75 (2.6 ± 0.6 MBq; 25.2 ± 6.3 μg; *n* = 8) or [^68^Ga]Ga-NOTA-non-specific Nb (1.4 ± 0.6 MBq; 21.2 ± 13.8 μg; *n* = 4) *via* the tail vein in tumor-bearing mice. For PET studies, energy window selected 300–700 KeV. CT acquisition was set to 50 kV at 300 μA, eight shots, 360 projections, and standard resolution. PET image reconstruction was accomplished using a 3D-OSEM (ordered subset expectation maximization) algorithm (21 subsets and five iterations), with random and scatter correction.

The whole-heart was delineated to perform blood kinetics of specific and non-specific nanobodies. Twelve dynamic frames were obtained over a 60 min acquisition time. A classical two-compartment pharmacokinetic model was applied to represent the disposition of the radiolabeled-nanobodies in the mice after a bolus injection. %ID/ml was computed by non-linear curve fitting performed in OriginPro 8 software.

Images were analyzed with the Biomedical Image Quantification Software PMOD (Version 4.302). Manually drawn volumes-of-interest (VOIs) were performed in tumors, heart, liver, and kidneys. These VOIs were selected from PET images using CT for anatomical limits. The radiotracer accumulation in organs was expressed in percentage of the injected dose per gram of tissue (%ID/g); the tumor maximum uptake (%ID/g_max_) was also calculated identifying the 20 connected pixels whose average value is maximal within the VOI. The maximum tumor-to-blood ratio (TBR) was determined for each tumor.

### Immunohistochemistry

Tumor-bearing mice were sacrificed after PET imaging and tumor were excised, fixed in 10% buffered formalin (Sigma, Saint Louis, MO) and embedded in paraffin. For histopathological analysis, tissues were serially sectioned (3 μm) and stained with hematoxylin and eosin (H&E). For analysis of MT1-MMP expression, immunohistochemical staining was performed using anti-MT1-MMP LEM 2/15 antibody at 1:400 dilution after antigen retrieval with low pH buffer in Autostainer platform (Dako, Santa Barbara, CA) and counterstained with hematoxylin. Whole slides were acquired with a slide scanner (AxioScan Z1, Zeiss, Jena). Regarding analysis, an appropriate script was created using Zen Blue Software (V 3.1, additional module for analysis, Zeiss, Jena).

### Statistical analysis

Statistical analysis was done using SPSS software package and a p value less than 0.05 was regarded as statistically significant in all assays. For *in vivo* experiments, mice were attributed to each group randomly at the beginning of the experiment. A one-way multivariate or univariate analysis of variance was run to determine the effect of radiolabeled-nanobodies injection on its pharmacokinetics and tumor uptake, respectively. Data are expressed as mean ± SD.

## Results

### Selection of membrane type 1-matrix metalloproteinas-specific nanobodies

**FIGURE 1:**
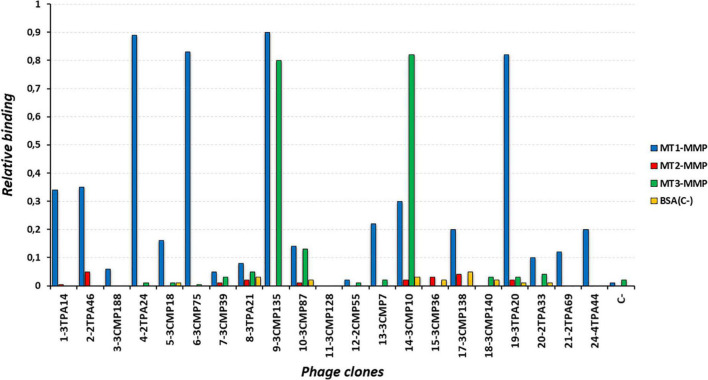
Reactivity of different clones representing most of the complementarity determining region 3 (CDR3) groups by phage ELISA against membrane type 1-matrix metalloproteinase (MT1-MMP) (blue bars) and other related members of the protein family, membrane-type matrix metalloproteinase subgroup, MT2-MMP (red bars) and MT3-MMP (green bars). Bovine serum albumin (BSA) (orange bars) was used as negative control. Number at the beginning of each phage clone denotes the CDR3 group at which it belongs.

**FIGURE 2:**
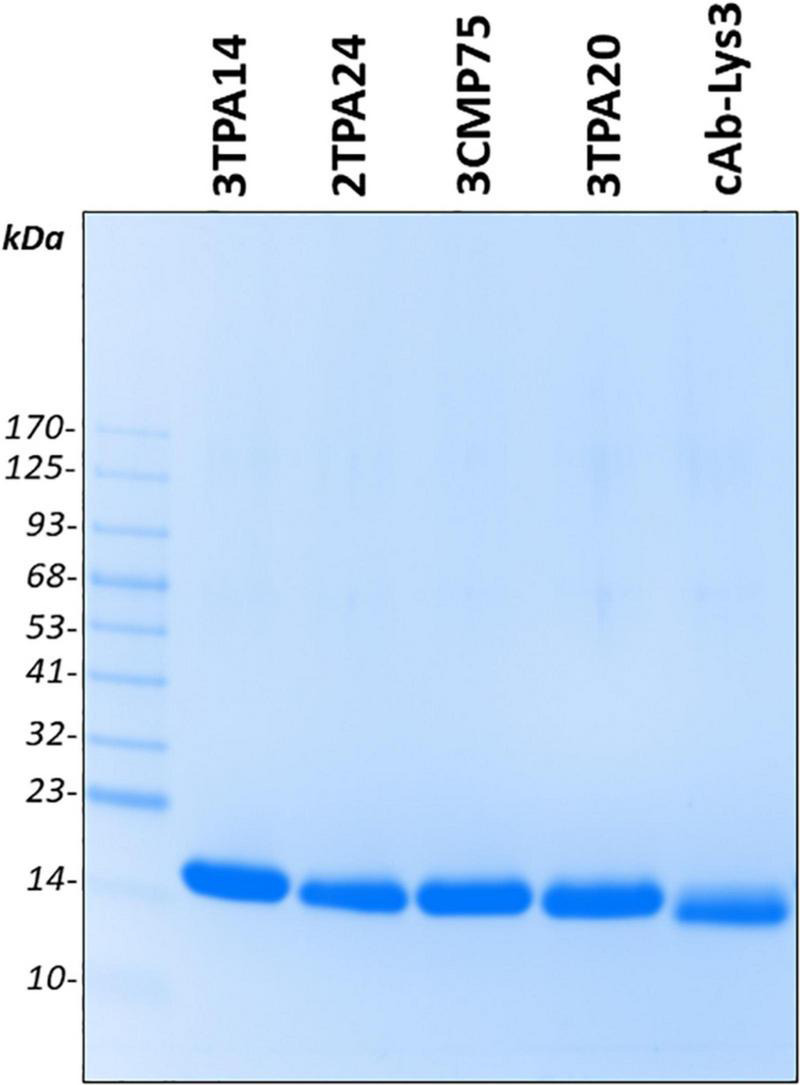
Purification of the selected anti- membrane type 1-matrix metalloproteinase (MT1-MMP) nanobodies. Sodium dodecyl sulfate-polyacrylamide gel electrophoresis (SDS-PAGE) analysis followed by Coomassie Blue staining in a 4–20% gradient polyacrylamide gel of purified nanobody clones 3TPA14, 2TPA24, 3CMP75, 3TPA20, and cAb-Lys3 as non-specific one. Periplasmic extract were subject to immobilized metal ion affinity chromatography followed by size exclusion chromatography in PBS buffer. Molecular weight markers are indicated at the left of the gel.

To develop nanobodies against MT1-MMP, a library of 2 × 10^9^ independent transformants was generated from a llama immunized with bacterial-derived CAT-MT1-MMP. The presence of folded MT1-MMP after refolding from inclusion bodies was verified by a cooperative thermal unfolding transition with a Ti of 62.7°C (Supplementary Figure 1). Following six rounds of immunization, lymphocytes were purified from their peripheral blood, the cDNA was synthesized by RT-PCR and VHH regions were cloned into phagemid vectors to generate a phage-display library. After four rounds of phage panning, 570 bacterial clones were picked randomly and the corresponding periplasmic extracts containing VHHs were analyzed for specific binding to CAT-MT1-MMP recombinant protein by ELISA. Out of these 570 colonies, 322 colonies were ELISA positive and consequently selected for DNA sequencing of the corresponding cloned nanobodies. Based on sequence data of the 322 positive colonies, 87 different nanobodies were identified, belonging to 24 different groups according to their unique CDR3 regions (B-cell lineages) (Table 1 and Supplementary Figure 2). Since nanobodies within one CDR3 cluster are expected to target the same epitope, 21 representative clones (one from each group, except groups 16, 22, and 23 due to their low reactivity against MT1-MMP) were selected to assess their specificity by a monoclonal phage ELISA against other closely related members of the membrane-type matrix metalloproteinase subgroup, MT2-MMP, and MT3-MMP. These assays revealed that 8 out of 22 clones were able to bind with high specificity to MT1-MMP but not to MT2-MMP or MT3-MMP (Figure 1) and therefore were selected for expression and purification of the corresponding VHHs. Those clones were: 3TPA14 from group 1, 3CMP188 from group 3, 2TPA24 from group 4, 3CMP18 from group 5, 3CMP75 from group 6, 3TPA20 from group 19, 2TPA69 from group 21, and 4TPA44 from group 24. Those eight Nbs were expressed in the non-suppressor *E. coli* WK6 strain, extracted from the periplasm by mild osmotic shock and purified by a two-step process using IMAC followed by size-exclusion chromatography (SEC). An irrelevant nanobody against lysozyme (cAb-Lys3) (24) was also produced as negative control for further comparative experiments. The yield of nanobodies 3TPA14, 2TPA24, 3CMP75, and 3TPA20 ranged from 1.8 to 9 mg per liter of bacterial culture (Supplementary Table 1) and the analysis on SDS-PAGE of each purified sample showed a single band migrating around 14 KDa with a purity of ≥95% (Figure 2), whereas nanobodies 3CMP188, 3CMP18, 4TPA44, and 2TPA69 were poorly overexpressed and making them difficult to purify, therefore they were discarded for further characterization.

**TABLE 1:** Nanobodies identifies against membrane type 1-matrix metalloproteinase (MT1-MMP) by phage display screening.

Group	Representative clones tested	Frequency	CDR3 sequence
1	3TPA14	27/87	TGYGRRSMPALRPEEWTY
2	2TPA8	8/87	NVVLRPGWVPRGY
3	3CMP188	7/87	NLRLSRGGDY
4	2TPA24	7/87	NARSFGDDY
5	3CMP18	5/87	NARRITAMGTTNDH
6	3CMP75	5/87	NQRNFGRDGTLGDY
7	3CMP39	5/87	AAGQHGTDY
8	3TPA21	4/87	AAKTASLGWLATMRRGQNDY
9	3CMP135	2/87	AARIGGYYYREGAYDY
10	3CMP87	2/87	ARVGGSWHLEV
11	3CMP128	2/87	AVVDPRDYGRVLFGS
12	2CMP55	1/87	AAHLIPYYSGPYYAMVPADFDS
13	3CMP7	1/87	AANPRWGNLLYDY
14	3CMP10	1/87	AVLTKYWG
15	3CMP36	1/87	AASEVGVTTTPSGYAY
16		1/87	NVRRVVADSIVDY
17	3CMP138	1/87	AQYGGGSPVPKWAA
18	3CMP140	1/87	AARSRTTYNLNNYYDY
19	3TPA20	1/87	NARKFRGPITDY
20	2TPA33	1/87	ARGGKYNYAD
21	2TPA69	1/87	NMRGSRLDY
22		1/87	NVRRRRYFGYDDY
23		1/87	AADPFAHYGNRPRSYAY
24	4TPA44	1/87	ARYVGNSGHYYKSSTS

### *In vitro* characterization of the anti-membrane type 1-matrix metalloproteinase nanobodies

**FIGURE 3:**
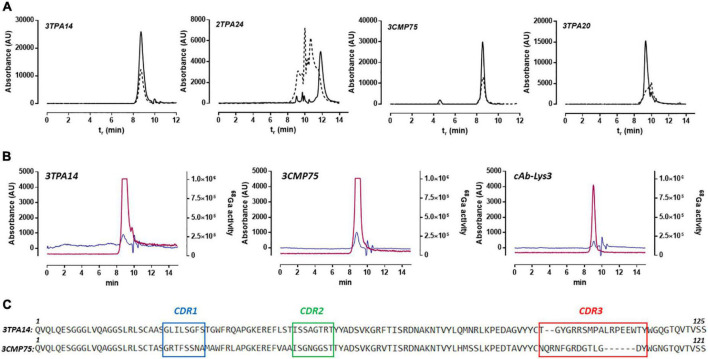
High-performance liquid chromatography-size exclusion (HPLC–SEC) profiles of nanobodies. **(A)** Chromatograms of absorbance at 280 nM for the selected nanobodies (solid line) and conjugated with NOTA chelator (dashed line). **(B)** HPLC chromatograms of [^68^Ga]Ga-NOTA-3TPA14, [^68^Ga]Ga-NOTA-3CMP75, and [^68^Ga]Ga-NOTA-cAb-Lys3 (non-specific) tracers; blue: 280 nM absorbance and red: activity of ^68^Ga. **(C)** Amino acid sequences of the finally selected nanobodies 3TPA14 and 3CMP75. The CDR1, CDR2, and CDR3 are boxed in blue, green and red, respectively. The first and last amino acids are numbered above each sequence.

For *in vivo* PET experiments, a further selection step was also performed based on critical quality attributes such as the presence of high/low-molecular weight fragments of nanobodies after conjugation with NOTA, which can be characterized using SEC in a high-resolution and high-throughput manner. Figure 3A shows an SEC separation of the selected nanobodies; the results showed that 3TPA14 and 3CMP75 nanobodies had minimal or no secondary interaction with the column since both nanobodies showed similar narrow chromatographic peak shapes and retention times (8.6 min). In contrast, 2TPA24 and 3TPA20 had deteriorated peak shapes with delayed retention times at high salt concentration mobile phase, indicative of potential hydrophobic interaction between analytes and the column; as these chromatographic profiles cannot be used to determine the radiochemical purity of the radiolabeled-Nbs, they were excluded from the experiments. Finally, two nanobodies, 3TPA14 and 3CMP75 (sequences in Figure 3C), were selected for radiolabeling and PET imaging.

Labeling of selected NOTA-nanobodies (3TPA14 and 3CMP75) and a non-specific NOTA-Nb with ^68^Ga resulted in overall radiochemical yields of 52.1 ± 7.6% (*n* = 10), 66.4% ± 10.6 (*n* = 9), and 37.8 ± 17.3% (*n* = 5), respectively, after PD-10 column purification. Radiochemical purity was always more than 99% (Figure 3B).

A binding assay (ELISA) was performed to confirm the preservation of the binding capabilities to CAT-MT1-MMP of the radiolabeled-nanobodies. 3TPA14 and 3CMP75 showed similar binding affinities between native antibodies and their corresponding radiolabeled-NOTA conjugates (Supplementary Figure 3). Therefore, 3TPA14 and 3CMP75 were finally selected as candidates for subsequent *in vivo* imaging studies.

**FIGURE 4:**
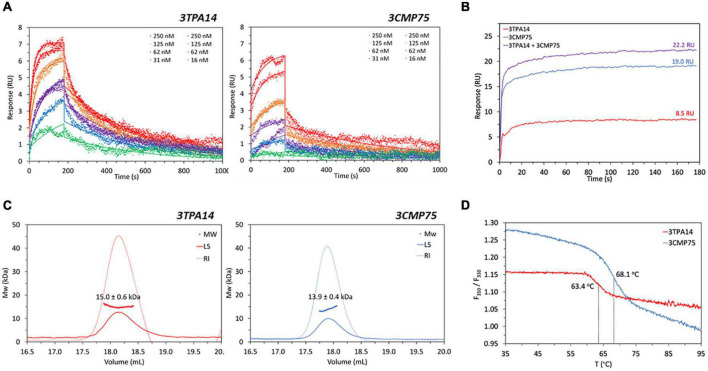
Biophysical characterization of the selected 3TPA14 and 3CMP75 nanobodies. **(A)** Surface plasmon resonance (SPR) titrations. Overlay of SPR sensorgrams and corresponding kinetic fits to a 1:1 binding model (solid line) of 3TPA14 and 3CMP75 nanobodies with MT1-MMP immobilized on a sensor chip. Replicate injections were done at 250, 125 and 62 nM nanobody concentrations. **(B)** SPR 3TPA14/3CMP75 competition. Overlay of SPR association sensorgrams showing absence of competition for the same MT1-MMP epitope between 3TPA14 and 3CMP75. Shown are the responses during three consecutive 3 min injections to immobilized MT1-MMP of 1.6 μM 3TPA14, 3.6 μM 3CMP75 and 1.6 μM 3TPA14 + 3.6 μM 3CMP75 mixture. **(C)** Size-exclusion chromatography combined with multiangle light scattering (SEC-MALS) Analysis. Chromatographic traces of light scattering at 90° (LS; solid lines), refractive index (RI; dashed lines) and the calculated molecular weight of the peaks from 3TPA14 and 3CMP75. **(D)** NanoDSF (Differential Scanning Fluorimetry) thermal unfolding profiles of nanobodies 3TPA14 (red line) and 3CMP75 (blue line). Following the change in fluorescence intensity ratio at 350 and 330 nM (F350/F330) with temperature, inflection temperatures of 63.4 and 68.1°C were determined for 3TPA14 and 3CMP75, respectively.

The affinity kinetics of the selected anti-MT1-MMP Nbs were determined by surface plasmon resonance (SPR) on immobilized recombinant MT1-MMP ectodomain. Both Nbs showed a high binding affinity, with K_D_ values in the nanomolar range, 15.8 ± 0.3 nM for 3TPA14 and 35 ± 3 nM for 3CMP75 (Figure 4A and Supplementary Material 1). To determine whether the Nbs recognize the same or different epitopes, the ability of the Nbs to compete with each other for MT1-MMP binding was also analyzed by SRP. Individual injections of 3TPA14 and 3CMP75 at 100-fold concentrations of the Nb K_D_ to saturate its epitope (1.6 and 3.6 μM, respectively) and a mixture of both at those same concentrations were performed over the chip with the immobilized protein and the responses were compared. Figure 4B clearly show that 3TPA14 and 3CMP75 recognized different (or overlapping) epitopes since the addition of the individual responses of each nanobody (8.5 + 19 RU) partially agreed with that of the 3TPA14/3CMP75 mixture (22.2 RU) after consecutive 3 min’ injections.

To complete the biophysical characterization, the two selected Nbs were analyzed by SEC-MALS in order to assess their homogeneity and size distribution under non-denaturing conditions, and by nanoDSF to examine their thermal stability which is critical for drug development, storage and delivery. MAL-SEC chromatograms displayed a unique symmetrical peak confirming sample purity and homogeneity in solution and molecular weight calculations based on RI and UV signals gave similar results as expected, 15.0 ± 0.6 KDa for 3TPA14 and 13.9 ± 0.4 KDa for 3CMP75 (Figure 4C). Finally, DSF-based studies revealed inflection temperatures (Ti) of 63.4 and 68.1°C for 3TPA14 and 3CMP75, respectively (Figure 4D), indicating a high stability in both cases.

### Pharmacokinetics and biodistribution of [^68^Ga]Ga-NOTA-nanobodies using PET/CT imaging

The diagnostic value of MT1-MMP specific Nanobodies was analyzed in mice bearing subcutaneous MDA-MB-231 xenograft of TNBC model.

**FIGURE 5:**
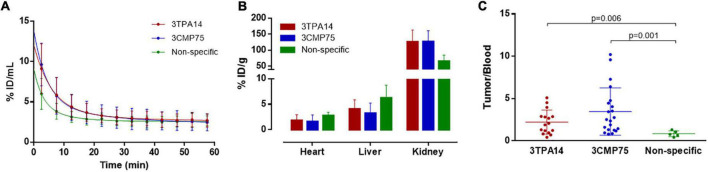
Biodistribution and pharmacokinetics of [^68^Ga]Ga-NOTA-Nanobodies. **(A)** Curves obtained from dynamic PET images representing the blood (heart left ventricle) concentration-time profiles from animals injected with [^68^Ga]Ga-NOTA-3TPA14 (red line), [^68^Ga]Ga-NOTA-3CMP75 (blue line), and [^68^Ga]Ga-NOTA-Non-specific (green line). **(B)** Biodistribution (%ID/g) of the radiotracers [^68^Ga]Ga-NOTA-3TPA14 (red bars), [^68^Ga]Ga-NOTA-3CMP75 (blue bars), and [^68^Ga]Ga-NOTA-Non-specific (green bars) in heart, liver, and kidneys. **(C)** Uptake levels of [^68^Ga]Ga-NOTA-3TPA14 (red dots), [^68^Ga]Ga-NOTA-3CMP75 (blue dots), and [^68^Ga]Ga-NOTA-Non-specific (green dots) in MDA-MB-231-tumors expressed as maximum tumor-to-blood ratios from static PET images at 45 min after iv injection of radiotracers. *P*-values are indicated. All data are represented as mean ± SD.

Firstly, we performed pharmacokinetics studies by calculating the blood kinetics of both specific nanobodies (3TPA14 and 3CMP75) and the non-specific one. The data obtained appeared to be similar in all cases, showing a fast decrease of blood concentration, followed by more progressive decline (Figure 5A). The non-specific Nb showed slightly slower blood clearance in comparison with the specific Nbs. (Supplementary Table 2).

Biodistribution results showed in general that the radiolabeled-Nb underwent rapid renal clearance accompanied by moderate hepatobiliary clearance. The accumulation of the radiotracers in the liver was (3.23 ± 2.00, 4.07 ± 1.78, and 6.24 ± 2.47% ID/g for 3CMP75, 3TPA14, and non-specific-Nb, respectively). In the kidneys, significantly higher uptake was observed for all radiolabeled-nanobodies (127 ± 34, 126 ± 37, and 65 ± 19% ID/g for 3CMP75, 3TPA14, and non-specific Nb, respectively), which suggested that the radiotracers were mainly cleared *via* the renal pathway (Figure 5B).

Finally, univariate ANOVAs showed that maximum tumor/blood ratio was statistically significant (*p* = 0.008) between injected nanobodies. Tukey *post-hoc* tests showed that [^68^Ga]Ga-NOTA-3CMP75 and [^68^Ga]Ga-NOTA-3TPA14 had statistically significant 4.1 and 2.6-times higher ratio, respectively (3.46 ± 2.80 and 2.21 ± 1.42; *p* = 0.001 and 0.006, respectively) than [^68^Ga]Ga-NOTA-non-specific Nb (0.84 ± 0.35) (Figure 5C).

**FIGURE 6:**
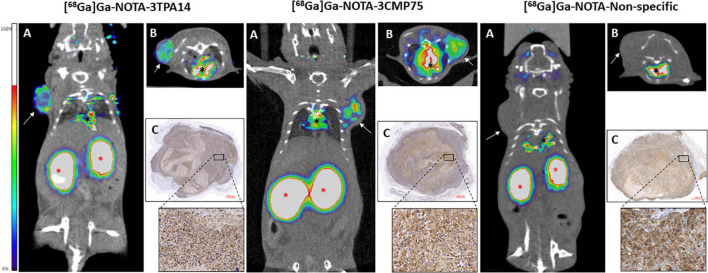
PET/CT images of the different radiolabelled nanobodies [^68^Ga]Ga-NOTA-3TPA14, [^68^Ga]Ga-NOTA-3CMP75, and [^68^Ga]Ga-NOTA-Non-specific performed in a triple-negative breast cancer (TNBC) mouse model showing their differences in tumoral uptake. Notice that the highest activity in tumour appears when PET-CT was performed with [^68^Ga]Ga-NOTA-3CMP75 probe (central panel). White arrows pointed the breast tumours in each mouse. Red asterisks pointed the kidneys and black asterisks, the hearth. **(A)** Coronal view, **(B)** axial view, and **(C)** immunohistochemistry using anti MT1-MMP LEM 2/15 Ab with a close-up showing high MT1-MMP expression. Red scale bars: 1,000 μm and close-up: 20 μm.

The tumor could be clearly visualized in PET images, indicating that both specific radiolabeled-nanobodies, in particular [^68^Ga]Ga-NOTA-3CMP75, can specifically target MT1-MMP (Figure 6 and Supplementary Video 1 for visualization with [^68^Ga]Ga-NOTA-3CMP75). Staining of the resected tumor tissue by immunohistochemistry with an antiMT1-MMP mAb demonstrated intensive membrane expression of this antigen on tumor cells in agreement with the imaging data (Figure 6C). Those data together indicated a relevant tumor targeting capability of the MT1-MMP-specific nanobodies for TNBC immunoPET imaging and the superior accuracy of the [^68^Ga]Ga-NOTA-3CMP75 probe in detecting TNBC by immunoPET.

## Discussion

Triple-negative breast cancer is an aggressive type of breast cancer with a high metastatic capability, a poor prognosis and high relapse rate within 5 years after treatment, up to 3-fold higher compared to non-TBC cases (28). These features make its efficient diagnosis of crucial importance for ensuring optimal management of the disease. Therefore, there is an urgent need for the development of real-time, accurate, non-invasive and advanced technologies to improve the diagnostic process in those patients. MT1-MMP plays an important pathogenic role in cancer growth, invasion and metastasis, and consequently it is becoming an emerging and appealing pharmacologic target for cancer diagnosis and treatment. Our group and others have previously developed some preclinical targeted-based approaches for PET imaging of MT1-MMP overexpression in glioma, pancreatic cancer, and bone sarcoma (17, 18, 29) based on the full-length IgG murine anti-MT1-MMP monoclonal antibody LEM2/15 (30) or by using radiofluorinated MT1-MMP substrate peptides conjugated to BODIPY650/665 and polyethylene glycol (PEG) for a dual PET/optimal imaging (31). Also, other full length IgG specific monoclonal antibody (113-5B7) coupled to fluorescent label rhodamineX, ^111^In-labeled miniaturized antibodies such as a scFv and a dimer of two molecules of scFv (diabody) and peptides conjugated with near-infrared fluorescent (NIRF) dye Cy5.5 or ^99m^Tc have been explored for monitoring MT1-MMP in several cancers by SPECT and optical imaging (32–35). Despite these studies, the development of novel probe formats with improved performance remains a challenging way to ameliorate cancer detection and to generate more accurate diagnosis in particular for TNBC. Several studies have demonstrated the utility of nanobodies in imaging and targeting tumors (20, 36–38) mainly due to their small size and half-life, low immunogenicity, stability and low-cost production. Also importantly, short half-life radionuclides, i.e., ^68^Ga (radioactive half-life 67.7 min) are required for radiolabeling to match its fast rapid blood clearance (39), allowing same-day imaging more convenient for patient comfort. The major milestones towards the clinical translation of nanobody-based PET probes are currently the phase II study of a ^68^Ga-NOTA-labeled anti-HER2 nanobody in HER2-positive breast cancer patients (40) and the phase I/II study of a [^68^Ga]Ga-NOTA-anti-MMR (Macrophage Mannose Receptor) for the detection of tumor-associated macrophages (TAM) (41). In the case of TNBC, nanobodies against tumor-specific antigens such as TNF-α, EGFR, CD3, CTLA-4, STAT-3, AKT2 among others, have been generated and tested for targeting cancer cells (22). Herein, we propose the development of MT1-MMP-specific nanobodies as candidates for TNBC diagnosis by non-invasive diagnostic imaging. To our knowledge, this is the first report of nanobody-based immunoPET imaging of MT1-MMP in breast cancers.

We generated 87 different llama-derived nanobodies, clustered in 24 groups according to CDR3 amino-acid sequence homology. After the screening processes based on cross-reactivity with other members of membrane-anchored metalloproteinases, efficient periplasmic expression and labeling stability, two final candidates were selected for a further characterization of their affinity, thermostability and targeting capability *in vivo*, 3TPA14 and 3CMP75.

*In vitro* analysis by Biacore showed that both nanobodies had nanomolar affinities for the MT1-MMP protein, 15,8 nM for 3TPA14 and 35 nM for 3CMP75, which are within the affinity range of other nanobodies (42, 43). By means of a competitive assay we could determine that the selected antibodies recognized two different epitopes on MT1-MMP although some interference of binding sites could be observed since the SPR signal of the 3TPA14/3CMP75 mixture (22.2 RU) was slightly lower than the addition of the individual responses (8.5 + 19 RU), which might be due to partial overlapping epitopes and/or to conformational effects. A major advantage of having nanobodies that bind to different epitopes would be the theoretical possibility of generating biparatopic constructs directed against MT1-MMP for improved potency and functionalities (44, 45).

Next, we initiated the search for an imaging probe by functionalizing nanobodies with NOTA in a random manner on exposed lysine residues and then radiolabeling with ^68^Ga. This strategy resulted in good radiochemical yields and excellent radiochemical purity. Given that this strategy could affect the binding capabilities of the nanobodies in case lysines were involved in epitope recognition, ELISA studies were performed to test the reactivity of conjugated and unconjugated nanobodies on MT1-MMP. The ELISA binding results for both nanobodies with or without NOTA conjugation showed an unchanged affinity and therefore this process did not provoke any effect on the binding of nanobodies to MT1-MMP. So, the feasibility of [^68^Ga]Ga-NOTA-3TPA14 and [^68^Ga]Ga-NOTA-3CMP75 for MT1-MMP detection was demonstrated. Nevertheless, lysine-based conjugation process used to yield heterogeneous conjugates, which are mixtures of species with different numbers of NOTA molecules linked at different sites on the nanobody sequence, so it would be worth testing if a site-specific strategy such as *via* sortase-A enzyme or cysteine-maleimide conjugation would improve the radiolabeling yields and performance of these nanobodies (16, 46).

The diagnostic value of [^68^Ga]Ga-NOTA-3TPA14 and [^68^Ga]Ga-NOTA-3CMP75 for TNBC detection by immunoPET was evaluated in the subcutaneous xenograft mouse model of the triple-negative breast cancer cell line MDA-MB 231 (47). A significantly higher uptake of MT1-MMP nanobody tracers was detected in tumors compared to a non-specific control nanobody tracer, confirming the specific targeting of anti-MT1-MMP nanobodies. A rapid clearance from muscle and blood was observed, resulting in a clear tumor visualization over the background at 45 min post-injection. Among them, [^68^Ga]Ga-NOTA-3CMP75 showed the highest targeting specificity and thus, becoming our lead candidate for further development. The biodistribution analysis showed that all the tracers tested were quickly cleared through the renal pathway which is a typical feature of nanobodies due to their small molecular weight (39). The renal uptake for the [^68^Ga]Ga-NOTA-non-specific Nb was lower than for the specific radiolabeled-nanobodies, most likely due to the lower blood clearance of the non-specific Nb as it has been described in the pharmacokinetic study.

One major drawback of nanobody-based PET is that long-term exposure to radiolabeled nanobodies can cause undesired adverse effects on kidneys. For clinical application, several strategies have been developed to reduce their renal retention without compromising the tumor uptake, for example, co-injection with gelofusine, lysine or monosodium glutamate to block the binding to megalin, a receptor responsible of the tubular reabsorption of nanobodies (48–50). Alternately, nanobodies can be modified by PEGylation to decrease renal uptake (51, 52). Another approach consists of removing the positively-charged hexahistidine affinity tag used during purification process, D’Huyvetter and collaborators demonstrated that polarity at the nanobody C-terminus can influence dramatically kidney retention, the highest accumulation occured with Myc-His-tagged anti-HER2 nanobody, followed by His-tagged and finally untagged nanobody (70–88% less accumulation) (49). With a view to clinical translation, future work will focus on further optimization and improvement of nanobody 3CMP75 to potentially enhance its activity as PET imaging probe, for instance, by testing site-direct radiolabeling and/or engineering an untagged format to decrease renal uptake. It is nevertheless noteworthy that this multi-objective probe optimization can remain a difficult, labor-intensive and time-consuming process that used to hinder imaging tool development (53).

In conclusion, we showed for the first time the *in vivo* evaluation of MT1-MMP expression in a TBNC mouse model using nanobody-based immunoPET imaging. Several llama-derived anti-MT1-MMP nanobodies were successfully generated and after an extensive *in vitro* characterization, two nanobodies were radiolabeled with ^68^Ga and their biodistribution profile was evaluated in mice xenografted with human TNBC cells. [^68^Ga]Ga-NOTA-3CMP75 showed the highest specific tumor uptake with an excellent tumor-to-blood ratio which make it a potential candidate for future clinical applicability in TNBC diagnosis.

## Data availability statement

The raw data supporting the conclusions of this article will be made available by the authors, without undue reservation.

## Ethics statement

The animal study was reviewed and approved by Competent Authority of the Regional Government of Madrid, Spain (project PROEX 294.8/20).

## Author contributions

FM, LF, MM, and JM-T conceived and designed the experiments. MO, GG, NM, LR, GM, CS, ER, RS, and YM performed the experiments. FM, RC-O, LF, MM, and JM-T analyzed the data. AA contributed to the reagents/materials. FM, MM, and JM-T wrote the manuscript. All authors contributed to the article and approved the submitted version.
