# Supplementary material for: Development of anti-membrane type 1-matrix metalloproteinase nanobodies as immunoPET probes for triple negative breast cancer imaging
Source: Front Med (Lausanne). 2022 Nov 24;9:1058455. doi: 10.3389/fmed.2022.1058455 (PMC9729729; doi:10.3389/fmed.2022.1058455)
Supplement: Supplementary file 1 [file Data_Sheet_1.pdf]

Supplementary Material

1 Supplementary Data

SPR data quality

SPR data quality was evaluated from the standard errors of the kinetic association (k_a) and dissociation (k_d) constants obtained from data fit to a 1:1 binding model. These errors were propagated to assess the error of the equilibrium/affinity constants (K_D), determined from the ratio of the rate constants. Moreover, as a measure of the goodness of fit, we examined: (1) χ^2 values, calculated as the average squared residual (the difference between the experimental data and the fitted curve) and (2) U-values, as an estimate of the uniqueness of the calculated values for the rate constants. When the U-value is below 15, the calculated parameter values are not significantly correlated and their absolute values, and not only their relative magnitudes, are meaningful. All these quality indicators are reported below.

Nanobody 3TPA14:
k_a = (1.64 ± 0.02) x 10^5 $M^{-1}s^{-1}$, k_d = (2.60 ± 0.02) x 10^{-3} s^{-1}, K_D = 15.8 ± 0.3 nM.
χ^2 = 0.071, U-value = 3.

Nanobody 3CMP75:
ka = (4.9 ± 0.2) x 104 $M^{-1}s^{-1}$, k_d = (1.73 ± 0.07) x 10^{-3} s-1, K_D = 35 ± 3 nM.
χ^2 = 0.048, U-value = 5.

Pharmacokinetics

The whole-heart was delineated to derive an image-derived blood input function. Twelve dynamic frames were obtained over a 60-minute acquisition time. Corresponding blood time-activity curves were generated by plotting the mean %ID/mL calculated in the blood versus time. A classical two-compartment pharmacokinetic model was applied to represent the disposition of the radiolabeled-nanobodies in the mice after bolus input and first-order elimination rate, described by the following equation:

$$\frac{\%ID}{mL} = A * e^{-alpha*t} + B * e^{-beta*t}$$

The initial estimates of A, B, alpha and beta parameters were computed by nonlinear curve fitting performed in OriginPro 8. The data were weighted by 1/Y, with Y as the predicted blood %ID/mL. Alpha and beta are constants that depend solely on k_{21}, k_{12} (transfer constants between central and peripheral compartments) and k_{10} (elimination constant); apparent terminal half-life ($t_{1/2,beta}$) is calculated as Ln2/beta. From these parameters, also several derived pharmacokinetic parameters can be calculated: Area Under the blood-concentration-time Curve (AUC), volume of distribution in the central compartment (V_C), terminal phase (V_Z) and at steady state (V_{SS}), and blood clearance (Cl).

The profile concentration vs time (Figure 5A) corresponded to a classical two-compartment pharmacokinetic model which consists of a rapid initial distribution phase followed by a progressive terminal elimination phase. The pharmacokinetic parameters are summarized in Table 2. Nanobodies given iv was characterized by a rapid disposition phase with a mean half-life of a few minutes (around

4 minutes) and a subsequent slower elimination phase with a mean terminal half-life of 2.2±1.4 h and 6.2±6.5 h for 3CMP75 and 3TPA14 nanobodies, respectively. Although the terminal half-life of 3TPA14 was 3-times higher than for 3CMP75, no significant statistically differences were found due to the high variability of the 3TPA14 terminal half-life. The blood concentration/time profile for the irrelevant nanobody was similar although the terminal half-life was higher 13.6±10.7 h than for the specific nanobodies; moreover, the initial blood concentration was lower for the irrelevant nanobody than for the specific nanobodies due its volume of distribution for the central compartment was higher than for the other nanobodies (13.02±4.37 mL vs 7.25±1.42 and 9.18±2.91 mL for 3CMP75 and 3TPA14, respectively). The AUC (area under curve) also increased for the irrelevant nanobody (3409±3027 %ID*min/mL vs 1719±1604 and 689±341 %ID*min/mL for 3CMP75 and 3TPA14, respectively) due to its slower total blood clearance (0.07±0.07 mL/min vs 0.19±0.11 and 0.11±0.09 mL/min for 3CMP75 and 3TPA14, respectively).

2 Supplementary Tables

Supplementary Table 1. Yield estimation for all eight purified selected nanobodies.

Nanobody clone	Group	Yield (mg/L of culture)
3TPA14	1	2.9
3CMP188	3	0.2
2TPA24	4	9
3CMP18	5	<0.1
3CMP75	6	4.5
3TPA20	19	1.8
2TPA69	21	0.3
4TPA44	24	<0.1

Supplementary Table 2. Mean pharmacokinetic parameters with their corresponding standard deviations (SD) calculated using a bicompartmental model after iv injection of [^{68}Ga]Ga-NOTA-Nanobodies

	3TPA14		3CMP75		Non-specific	
	Media	SD	Media	SD	Media	SD
A (%ID/mL)	8.75	3.65	10.68	2.70	5.76	2.77
B (%ID/mL)	3.25	0.86	3.61	1.49	2.75	0.65
alpha (min^{-1})	0.17	0.04	0.24	0.17	0.22	0.07
beta (min^{-1})	0.004	0.003	0.007	0.004	0.002	0.002
t_alpha (min)	4.2	1.0	3.6	1.3	3.6	1.7
t_beta (h)	6.19	6.53	2.21	1.36	13.58	10.69
Co (%ID/mL)	12.00	4.09	14.30	2.94	8.52	3.41
Vc (mL)	9.18	2.91	7.25	1.42	13.02	4.37
k21 (min^{-1})	0.05	0.02	0.06	0.04	0.07	0.02
k10 (min^{-1})	0.01	0.01	0.03	0.02	0.01	0.01
k12 (min^{-1})	0.11	0.03	0.16	0.12	0.14	0.05
AUCinf (%ID*min/mL)	1719	1604	689	341	3409	3027
Vss (mL)	29.04	8.65	26.04	8.90	36.67	8.58
Vz (mL)	30.71	8.36	28.53	9.85	37.23	8.55
Cl (mL/min)	0.11	0.09	0.19	0.11	0.07	0.07

2.1 Supplementary Figures

Supplementary Figure 1. NanoDSF (Differential Scanning Fluorimetry) thermal unfolding profile of the catalytic domain of human MT1-MMP (residues 119-290) (CAT-MT1-MMP) at 2 µM in HBS-EP+ buffer (10 mM HEPES pH 7.3, 150 mM NaCl, 3 mM EDTA and 0.05% P20). Following the change in fluorescence intensity ratio at 350 and 330 nm (F350/F330) with temperature, an inflection temperature (Ti) of 62.7 ºC was determined

Supplementary Figure 2. Reactivity by ELISA of 87 isolated VHH clones belonging to 24 different CDR3 groups (B-cell lineages). Measurements were expressed as ratio of absorbances at 450 nm obtained with MT1-MMP and a negative control.

Supplementary Figure 3. Reactivity by ELISA of ^{68}Ga-radiolabeled and non-radiolabeled nanobodies against MT1-MMP. **A)** Percentage of absorbance at 450nm of 3TPA14 (blue line and dots) and ^{68}Ga-3TPA14 (red line and dots). **B)** Percentage of absorbance at 450nm of 3CMP75 (blue line and dots) and ^{68}Ga-3CMP75 (red line and dots).

2.2 Supplementary Videos

Supplementary Video 2. 3D rendering of a mouse xenografted with MDA-MB231 cells by PET/CT with the MT1-MMP-specific [^{68}Ga]Ga-NOTA-3CMP75 tracer showing high uptake in the TNBC tumor.
