# Supplementary figures and images for: Development of anti-membrane type 1-matrix metalloproteinase nanobodies as immunoPET probes for triple negative breast cancer imaging
Source: Front Med (Lausanne). 2022 Nov 24;9:1058455. doi: 10.3389/fmed.2022.1058455 (PMC9729729; doi:10.3389/fmed.2022.1058455)

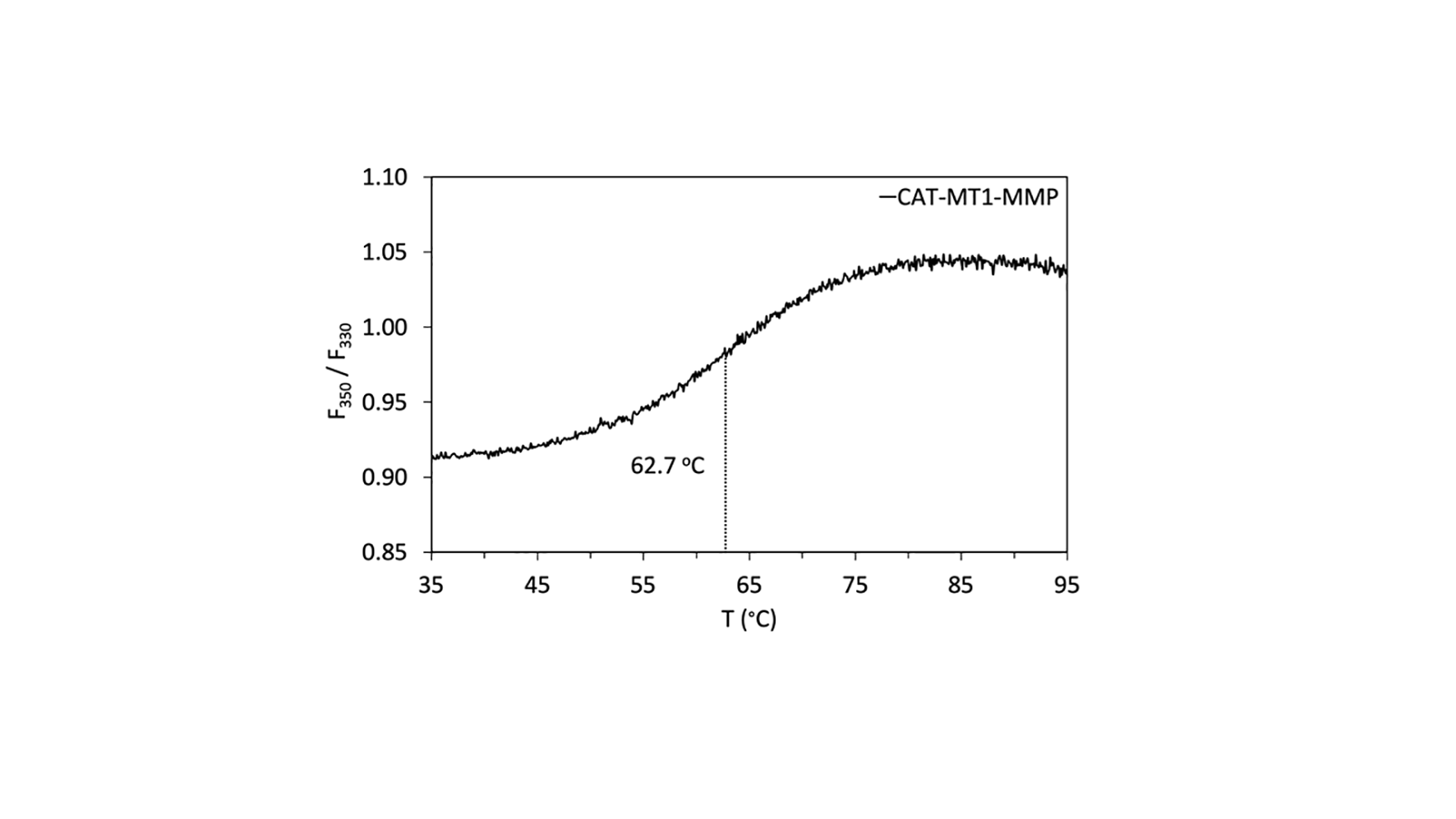

Supplement: Supplementary file 2 [file Image_1.TIF]

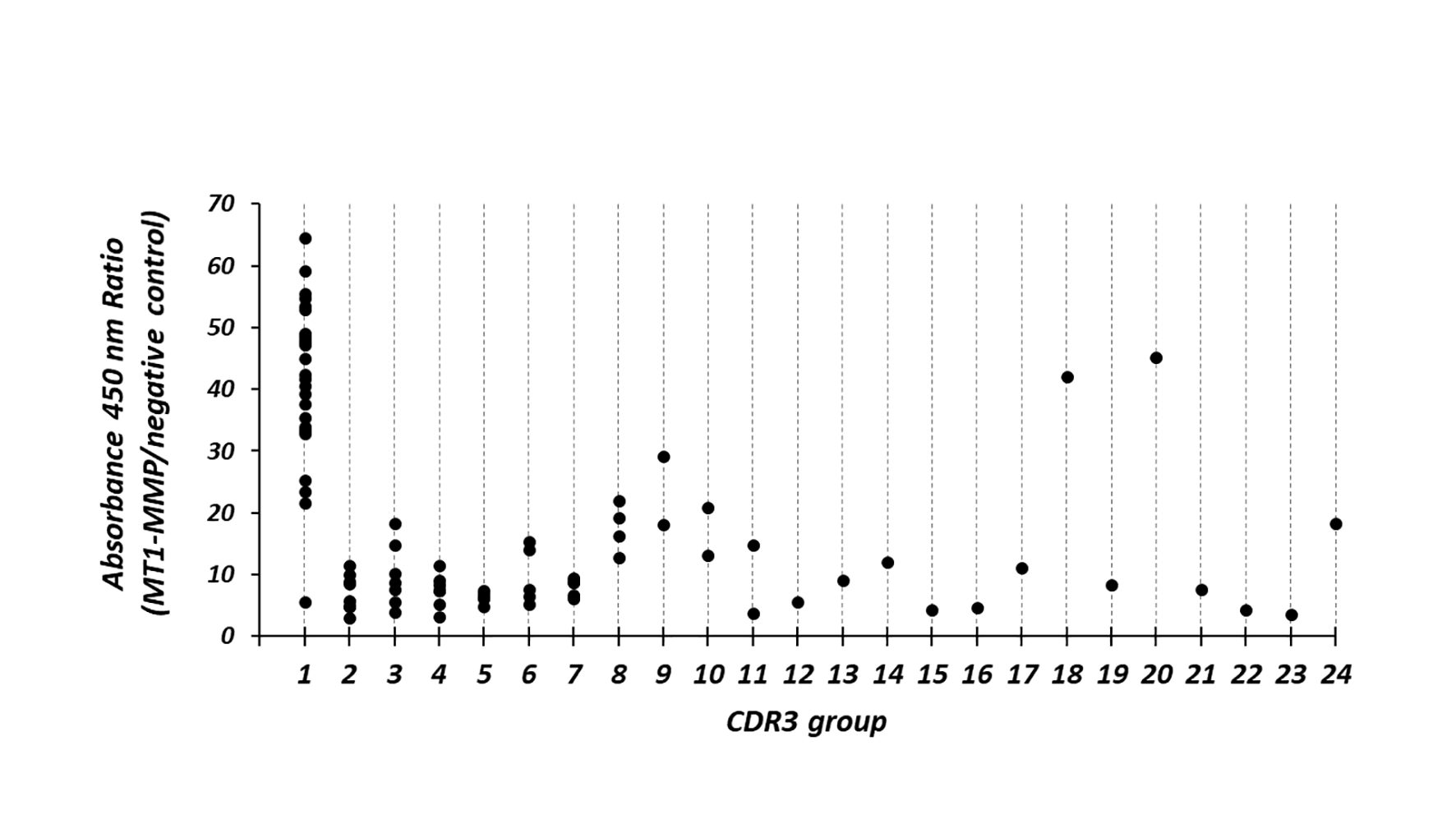

Supplement: Supplementary file 3 [file Image_2.TIF]

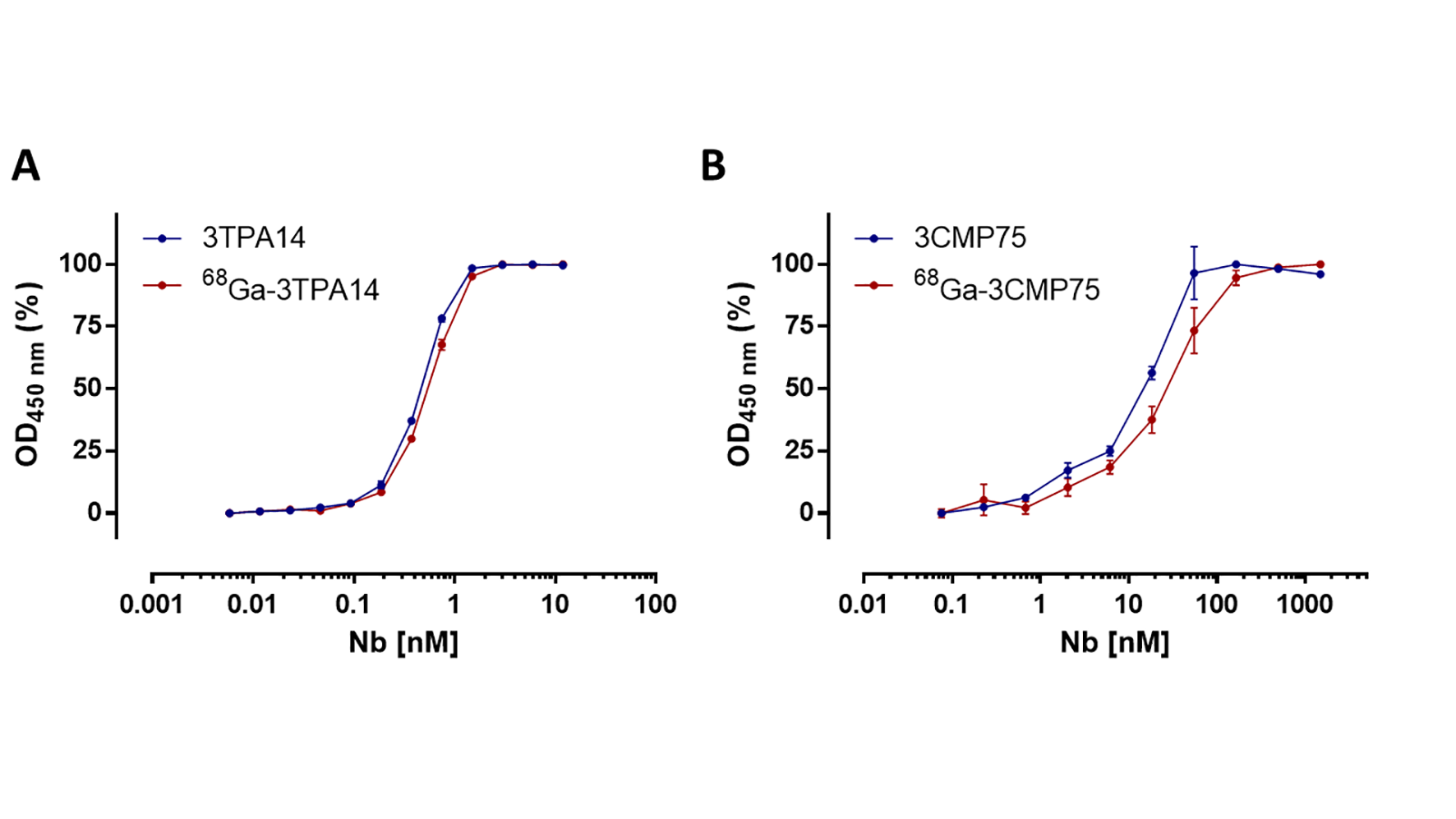

Supplement: Supplementary file 4 [file Image_3.TIF]
